# Difference between self-reported adherence to standard precautions and surveillance and factors influencing observed adherence: a quantile regression approach

**DOI:** 10.1186/s12912-022-00984-1

**Published:** 2022-07-25

**Authors:** Jin Suk Kim, Eunhee Lee

**Affiliations:** 1 Hallym University Medical Center Kangnam, Seoul, Korea; 2 School of Nursing, Hallym University, 1 Hallymdaehak-Gil, Chuncheon, Gangwon-do 24252 Republic of Korea

**Keywords:** Standard precautions, Healthcare-associated infections, Surveillance, Nurse staffing, Hand hygiene

## Abstract

**Background:**

Standard precautions are minimum healthcare-associated infection prevention practices applied in all healthcare settings. The aim of this study was to investigate adherence to standard precautions using a survey and surveillance. Factors affecting observed adherence to standard precautions were also determined.

**Methods:**

This cross-sectional observational study included 163 clinical nurses who were directly involved in patient care. Differences in adherence according to investigative methods are represented as a boxplot. Quantile regression was used to identify factors affecting observed adherence, including organizational factors (such as department, safety environment, and patient safety climate) and personal factors (such as knowledge and awareness). Stata SE version 14.2 was used for all statistical analyses.

**Results:**

The observed adherence to standard precautions was 76.8 out of 100, whereas the self-reported adherence was approximately 95. Hand hygiene adherence received the lowest score of less than 70. Factors influencing observed adherence were self-reported adherence (*p* = 0.043) in 25% and 50% quantiles, work experience (*p* = 0.002) in the 25% quantile, and working department (*p* = 0.030) in the 50% quantile. There were no significant factors in the 75% quantile.

**Conclusion:**

Inadequate adherence to standard precautions might increase healthcare-associated infections. Thus, an organizational environment such as nurse staffing needs to be established so that clinical nurses with high competency can comply with standard precautions in clinical settings.

## Background

The entire world is currently facing a crisis due to the outbreak of the novel coronavirus. The outbreak of infectious diseases has a great impact on society at large, including healthcare and the economy [1]. South Korea suffered extensive damage due to the Middle East Respiratory Syndrome (MERS) outbreak in 2015. Thus, the importance of infection control has been emphasized in amendments to infection-related laws and regulations [2].

Hospitals are among environments that are most vulnerable to infectious diseases because patients are susceptible to infections not only due to their age and comorbidities, but also due to surgeries and/or invasive procedures they undergo in the hospital [3]. The average prevalence of healthcare-associated infections [HAIs] has been estimated to be 4.5 – 15.5 per 100 patients, showing large differences by country, especially between Europe or the United States and developing countries [3, 4]. In addition, the fatality rate of HAIs has been reported to be 5.7% [5]. HAIs could affect several healthcare outcomes and significantly prolong the length of stay by 5 – 9 days [6, 7], resulting in significant increases of medical expenses [6–8]. Therefore, in a situation where the occurrence of new infectious diseases continues to increase, healthcare providers are trying to reduce and prevent HAIs. Despite various prevention efforts, more than one million patients experience HAIs annually in the United States and Europe [1, 9]. Since the MERS outbreak in 2015, South Korea has established and applied more proactive policies and strategies to prevent HAIs. However, HAIs remain a challenge to overcome.

Standard precautions [SPs] are minimum HAI prevention practices that should be applied proactively in all healthcare settings [10, 11]. The World Health Organization [WHO] also recommends healthcare providers to comply with SPs as a sustainable and widely applicable basic strategy to prevent HAIs [12]. Most countries have adopted SPs for national health policy infection control. SPs for all patient care include the following eight strategies: 1) hand hygiene, 2) the use of personal protective equipment (PPE), 3) safe injection practices, 4) cough etiquette, 5) environmental infection control including disinfection and sterilization, 6) handling textiles and laundry carefully, 7) isolation precautions, and 8) healthcare worker safety [13].

Several previous studies have reported that SP strategies are effective in preventing HAIs. Among them, hand hygiene has already been revealed as a basic and effective way to reduce HAIs [14, 15]. Furthermore, using an appropriate type of PPE in the right way can significantly prevent the spread of HAIs between patients [16]. Moreover, safe injection practices can greatly reduce the risk of blood-related infections, which can be fatal to patients [17]. Among different healthcare workers, nurses perform these three safety precautions frequently. In addition, since nurses stay with patients at the bedside and provide direct patient care from fundamental care to invasive care, adherence to SPs for clinical nurses has a great impact on HAI prevention. Despite the importance of nurses' adherence to SPs, it has been reported that their level of adherence is insufficient [14, 18–20].

Nurses’ adherence to SPs is influenced by both personal factors and environmental factors. Among personal factors, previous studies have reported that knowledge and awareness can significantly influence the adherence of nurses to SPs [10, 21–23]. To improve knowledge and awareness, education and training have been developed and implemented in clinical settings [11]. In the case of environmental factors, physical circumstances such as a heavy workload and the lack of resources and a safe environment are major factors faced by nurses [24, 25]. Recently, it has been reported that not only the physical environment, but also patient’s safety environment can influence nurses’ adherence to SPs [26]. A better patient safety climate is also related to greater adherence to SPs in various types of healthcare settings [26].

Although many previous studies have investigated factors influencing adherence to SPs, a limitation of several studies was that only self-reported adherence, not observed adherence, to SPs was investigated. In addition, adherence to SPs showed differences in results depending upon the investigation method and the observed adherence to SPs showed a tendency to be lower than self-reported adherence [18, 27–29]. Since whether self-reported adherence indicating intention is linearly associated with actual adherence has not been determined, we cannot consider self-reported adherence as actual adherence.

Therefore, this study investigated the following research questions:

- What is the difference between nurses’ self-reported adherence to SPs and observed adherence?
- Which personal factors (such as knowledge and awareness of SPs) and organizational factors (such as work department, safe environment, and patient safety climate) influence observed adherence to SPs?

## Methods

### Study setting and sample

This cross-sectional observational study conducted both a survey and a surveillance to examine and compare adherence of clinical nurses to SPs according to the investigative method. The sample size was calculated based on ordinary least squares (OLS) regression considering a significance level of 0.05, a median effect size, and a power of 0.80. We confirmed the acceptable power under a given sample size and effect size in quantile regression [30]. We included 163 clinical nurses who worked at one general hospital in South Korea. As this study aimed to investigate adherence to SPs, we only included nurses directly caring for patients, excluding nurse managers and nurses working in departments not directly related to patient care. This study included nurses who worked in various departments, not only in general care units (such as the general ward and the integrated nursing unit), but also in special care units such as the intensive care unit (ICU) and emergency room (ER). Integrated care units were implemented in 2013 in South Korea to decrease the caregiving burden and improve the quality of care. This unit provides care services by a nursing staff increased to about twice that of a general ward.

### Data collection

From August 2018 to September 2018, data on adherence to SPs were collected through a survey and a surveillance using an instrument developed by referring to the Centers for Disease Control and Prevention (CDC) guidelines [13]. The survey was conducted in various departments, ranging from general ward to ICU. First, we obtained written informed consent from participants. Data were then collected using structured questionnaires, which included adherence to SPs and related factors. To reduce external effects on observation as much as possible, we conducted an observational study more than one week after the survey was completed. In the observational study, to establish validity, we assigned three nurses working in infection control departments who received specialized training in the surveillance, prevention, and control of HAIs as observers. These three observers were trained several times on observational behavior and observation points using the developed instrument. Lastly, before the observational study, 20 cases were pre-observed using the instrument, confirming the instrument’s validity. Among these 20 cases, evaluation by the three observers was consistent for 17 cases, indicating moderate agreement (Fleiss’s kappa = 0.5148). The observers visited the unit only during the day shift on weekdays and observed nurses who completed the survey from a distance and evaluated the nurses’ behavior corresponding to each item on the developed instrument.

### Survey tool

Adherence to SPs was measured using the instrument developed according to CDC guidelines. Among eight SP strategies, three strategies (i.e., hand hygiene, safe injection practices, and the use of PPE while providing patient care directly related to patient care) were included in this study. The instrument on SP adherence consisted of five items for each of these three SP strategies for a total of 15 items. Each item was a question on a situation in which SPs should be performed. Circumstances in which handwashing should be performed included the following: 1) before touching a patient, 2) before cleaning/aseptic procedures, 3) after body fluid exposure risk, 4) after touching a patient, and 5) after touching patient surroundings. Hand hygiene included both hand washing with water and soap and hand rubbing with alcohol-based hand sanitizer [12]. Items on safe injection practices included the following: 1) disinfecting the rubber septum on a medication vial with alcohol before piercing, 2) not using needles or syringes for more than one patient, 3) discarding used infusion sets, 4) not using single-dose medication for more than one patient, and 5) using a new needle/syringe even when withdrawing additional doses for the same patient. Last, items of PPE included the following: 1) wearing a gown if clothes were soiled with body fluids, secretions, or excretions, 2) changing PPE after use for each patient, 3) removing a gown before leaving the patient’s room, 4) turning the contaminated outer part of the gown toward the inside when removing it, and 5) not reusing a gown.

The frequency of adhering to SPs was investigated in the survey with a 5-point Likert scale (5: always, 4: often, 3: sometimes, 2: rarely, and 1: never). Cronbach’s alpha was 0.88 in this study. In addition, the observational study evaluated whether nurses adhered to the SPs of 15 items in the developed instrument by observing whether or not they were performed. Observed adherence was calculated as the percentage performed out of 15 items. To compare differences in the distribution according to research methods, the self-reported adherence based on a 5-point scale was converted to a 100-point scale.

We determined four variables associated with adherence to SPs, i.e., knowledge, awareness, a safe environment, and patient safety climate derived from several previous studies [10, 23, 26]. Among these four variables, knowledge, awareness, and a safe environment were measured in this study by modifying the instrument developed by Jo and Choi [31] according to CDC guidelines [13]. Knowledge consisting of 25 items was measured by yes (1 point) or no (0 point) answers, with a total score ranging from 0 to 25. Awareness consisting of five items was measured on a 3-point Likert scale (2: highly agree, 1: agree, and 0: disagree), with total scores ranging from 0 to 10. The safe environment variable consisting of seven items was measured by yes or no, with a total score ranging from 0 to 7.

The patient safety climate was measured using an instrument developed by Moon [32], which consisted of 10 items scored on a 7-point Likert scale. It was evaluated by the average score. Cronbach alpha for the patient safety climate instrument was 0.88 in this study. Permission to use each instrument was obtained from each author.

### Ethical consideration

This study received ethical approval from Hallym University Medical Center Kangnam (HKS2018—06–017). Written informed consent was obtained from each participant. Permission to use the survey instrument was obtained by the primary investigator. We offered compensation to participants and observers of this study.

### Analysis

Nurses’ characteristics are reported as mean and standard deviation [SD] for continuous variables and frequencies and percentages for categorical variables. Descriptive analysis of the two types of adherence (self-reported and observed) and the four influencing variables are reported as mean and SD. After making the measurement level of the observed adherence and the self-reported adherence the same, the difference between the mean and distribution of the adherence according to the survey method was presented as a box plot. Correlations between the four influencing variables and the two types of adherence were analyzed using Pearson’s correlation coefficient. Quantile regression analysis was used to identify factors affecting observed adherence to SPs, assuming that variables influencing each adherence quantile were different and the observed adherence had a skewed shape and data outliers. Results from quantile regression and standard linear regression were compared. Stata SE version 14.2 was used for all statistical analyses.

## Results

A total of 163 nurses were included in this study. Their characteristics are shown in Table 1. Their average age was 27.5 years. Most (92.6%) nurses were women. More than 90% of these nurses had a bachelor’s degree or a diploma. Their average work experience was 54.9 months. Newly graduated nurses who worked less than a year accounted for 23.3% of participants in this study. Most (95.1%) nurses had received education on infection control.

**Table 1:** Characteristics of clinical nurses (*N* = 163) enrolled for this study

Characteristics	Categories	N (%)	Mean ± SD
Gender	Men	12 (7.4)	
	Women	151 (92.6)	
Age (years)	< 26	81 (49.7)	27.5 ± 5.5
	26 ~ 30	51 (31.3)	
	> 30	31 (19.0)	
Education level	Diploma	48 (29.4)	
	Bachelor’s	105 (64.5)	
	Master’s	10 (6.1)	
Work experience (months)	< 12	38 (23.3)	
	12 ~ < 36	54 (33.1)	54.9 ± 66.7
	36 ~ < 60	22 (13.5)	
	≥ 60	49 (30.1)	
Work department	Intensive care unit	50 (30.9)	
	Integrated care unit	13 (8.0)	
	Emergency room	19 (11.7)	
	General ward	80 (49.4)	
Education on infection control	Yes	155 (95.1)	
	No	8 (4.9)	

**Fig. 1:**
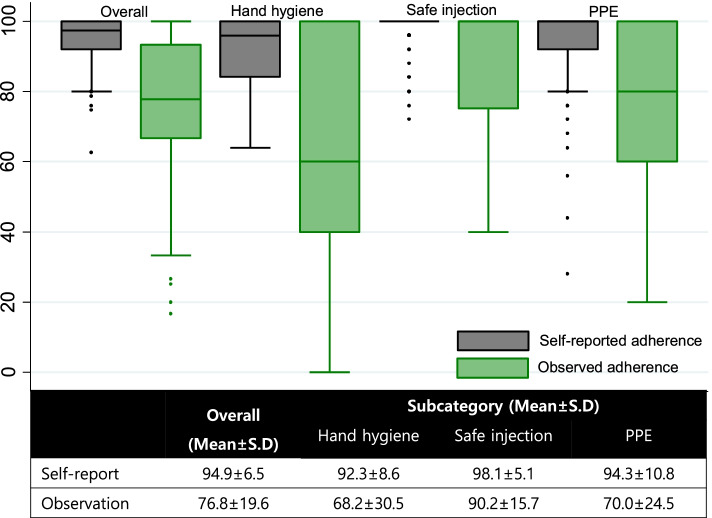
Comparison between self-reported adherence to SPs and observed adherence to SPs

Table 2 shows levels of four variables associated with adherence to SPs according to study methods of survey and surveillance. The average score was 21.5 for knowledge, 8.8 for awareness, 5.9 for patient safety climate, and 6.1 for safe environment. In the case of adherence to SPs, the self-reported adherence score was 4.7 points, which was approximately 95 points after converting it to a 100-point scale. In contrast, the observed adherence to SPs was 76.8 points, which was lower than the self-reported adherence (Fig. 1). Hence, adherence to SPs varied considerably depending on the investigation method. These surveillance results were lower than survey results not only for overall scores, but also for each strategy. According to each SP strategy, the difference between self-reported and observed adherence scores was the lowest for safe injection practices, which was 90 or higher in both methods. However, hand hygiene and the use of PPE showed differences of 20 points or more between self-reported and observed scores. The observed adherence to hand hygiene was under 70 points, which was the lowest among SPs.

**Table 2:** Levels of knowledge, awareness, patient safety climate, safe environment, and adherence to standard precautions

Variables	Items	Mean ± SD	[Min—Max]
Knowledge	25	21.5 ± 1.9	[14.0—25.0]
Awareness	5	8.8 ± 1.2	[5.0–10.0]
Safe environment	7	6.1 ± 1.0	[2.0- 7.0]
Patient safety climate	10	5.9 ± 0.8	[2.3—7.0]
Self-reported adherence	15	4.7 ± 0.3	[3.1—5.0]
Observed adherence	15	76.8 ± 19.6	[16.7—100.0]

Among the four variables related to the adherence to SPs, all variables except knowledge were significantly correlated with self-reported adherence. However, observed adherence was significantly correlated with only patient safety climate (*r* = 0.16, *p* = 0.036). In addition, there was a significant correlation between results of the two methods (*r* = 0.28, *p* < 0.001).

In the case of observed adherence to SPs, the average score was lower than self-reported scores and the distribution was larger than self-reported scores. Observed adherence ranged widely from 16.7% to 100%. The distribution of the observed adherence to SPs showed a skewed shape. Therefore, we investigated factors associated with observed adherence to SPs using quantile regression. To prevent multicollinearity, we included work experience instead of age. In addition, variables that showed significant correlations with observed adherence to SPs in Table 3 were selected as independent variables. There was no multicollinearity in the regression model because the variance inflation factor (VIF) was less than the threshold value [1.21 – 1.90].

**Table 3:** Correlations between knowledge, awareness, safe environment, patient safety climate, and adherence to standard precautions

Variables	r (*p-value*)					
	1	2	3	4	5	6
1. Knowledge	1					
2. Awareness	-0.15 (0.053)	1				
3. Safe environment	0.15 (0.057)	0.18 (0.023)	1			
4. Patient safety climate	0.25 (0.002)	0.33 (< 0.001)	0.45 (< 0.001)	1		
5. Self-reported adherence	0.10 (0.227)	0.23 (0.003)	0.41 (< 0.001)	0.57 (< 0.001)	1	
6. Observed adherence	0.06 (0.450)	-0.01 (0.973)	0.13 (0.091)	0.16 (0.035)	0.28 (< 0.001)	1

In ordinal least square (OLS) analysis, statistically significant variables were self-reported adherence, work experience, and work department. Regarding each quantile, statistically significant variables were self-reported adherence (*p* = 0.043) and work experience (*p* = 0.002) in the 25% quantile and self-reported adherence (*p* = 0.032) and work department (*p* = 0.030) in the 50% quantile. However, there were no significant variables associated with observed adherence in the 75% quantile (Table 4). In the case of the work department, the number of patients nurses were responsible for was the lowest in the ICU, followed by those in the integrated care unit, ER, and the general ward. In the regression model, adherence of nurses in the general ward was significantly lower than that of nurses in the ICU. Hence, if nurses take care of many patients, the actual adherence to SPs will inevitably decrease, especially in the middle adherence quantile.

**Table 4:** Factors influencing observed adherence to standard precautions using quantile regression analysis

Variables		OLS Regression		QR at 0.25 quantile		QR at 0.50 quantile		QR at 0.75 quantile	
		Coeff	SE	Coeff	SE	Coeff	SE	Coeff	SE
**Personal factors**	Self-reported adherence	11.91*	5.36	16.23*	8.18	13.62*	6.30	13.61	7.62
	Education on infection control	1.06	6.60	-9.92	22.38	-9.83	8.11	-1.15	9.46
	Work experience (years)	1.40**	0.35	1.86**	0.47	1.17**	0.38	0.72	0.51
	Education level (base: diploma)								
	Bachelor’s	-2.29	3.21	-4.04	5.26	-2.00	3.47	0.58	3.83
	Graduate	-4.53	7.86	-13.89	7.03	-2.03	11.65	-0.70	8.79
	Gender (base: men)	3.42	5.99	7.21	14.39	6.58	10.99	-1.27	7.49
**Organizational factors**	Patient safety climate	-0.58	2.28	-0.39	4.00	-0.94	2.18	1.63	3.27
	Work department (base: ICU)								
	Integrated care unit	2.27	5.85	-1.98	8.49	-2.49	5.00	-1.36	9.80
	Emergency room	0.17	5.18	1.39	7.68	-2.22	6.31	3.36	6.01
	General ward	-7.52*	3.68	-11.03	5.81	-8.43*	3.86	-2.32	5.67
**Constant**		18.34	23.23	-6.94	38.34	23.37	36.88	14.78	27.74
**R**^**2**^		.18		.15		.15		.13	

^*^p < 0.05, **p < 0.01

## Discussion

This study investigated adherence to SPs using a survey and a surveillance. The self-reported adherence score investigated by the survey was 4.7 points out of 5 points, meaning that most nurses responded that they always performed (5 points on the Likert scale) or often performed (4 points on the Likert scale) all SP items. These results were slightly higher than those of previous studies [10, 23]. However, results from previous studies were also high, reporting around 4 out of 5 points [10, 23]. This might be attributed to the importance of infection control emphasized in overall healthcare settings after the MERS outbreak in South Korea. These excessively high results might also be attributed to the investigation method.

In this study, the observed and self-reported adherence to SPs showed a large difference. Moret, Tequi, and Lombrail [33] have reported that differences between self-reported adherence and observed adherence are insignificant. They suggested the applicability of the self-reported method, which is easy to use and inexpensive. However, several studies have reported differences in SP adherence according to the investigation method [18, 27]. Poor validity of the self-reported method has also been reported [28]. Eldridge et al. [18] have investigated effects of hand hygiene practices and found that the observed adherence is increased significantly to 47% – 80%, whereas the self-reported adherence shows little change at the level of 87% regardless of hand hygiene practices. This means that self-reported adherence does not indicate actual performance or reflect clinical situations. Since healthcare providers regard adequate adherence to SPs as the healthcare provider’s obligation, self-reported adherence, which measures the intention to adhere to SPs, might be higher than actual adherence. Especially in the pandemic situation with excessive workloads and insufficient resources, adherence to SPs has been found to be lower than that in usual situations [34]. Thus, the gap between self-reported and observed adherence might be greater than results of this study depending on the clinical situation.

Among SP sub-strategies, although hand hygiene is a basic infection control practice, the observed adherence to hand hygiene was the lowest [below 70 points] among all SP strategies. Previous studies have also shown that the observed adherence to hand hygiene is low [14, 19]. In the case of no observers such as in video surveillance, the adherence is even worse [20]. Hence, real hand hygiene adherence might be extremely low because results excluding observer’s effect would be more realistic. Poor hand hygiene is a risk factor for HAIs [14, 15]. Therefore, studies investigating barriers hindering the adherence of nurses to hand hygiene and effective strategies to improve adherence are needed. The observed adherence to PPE use was much lower than the self-reported adherence. Their difference between the two methods was as large as that for hand hygiene. Compared to hand hygiene and safe injection practices, PPE use is affected more by external factors such as equipment shortages and nursing workload [34]. Thus, the observed adherence to PPE use in this study might have been affected by working conditions on the surveillance day. Among SP strategies, adherence to safe injection practices had the highest self-reported and observed scores. Since safe injection practices have the most direct effect on patients, scores should be increased as close to 100 points as possible by implementing various strategies.

As mentioned earlier, healthcare providers recognize the importance of perfect adherence to SPs and have a high intention to comply, consistent with results of this study. Hence, their low performance can never be attributed to low knowledge or low awareness. Other variables or barriers besides knowledge or awareness should be identified. Recently, some studies have reported that organizational conditions such as overcrowding and emergencies are factors hindering the adherence to SPs by clinical nurses [34, 35]. We conducted an observational study during the day shift on weekdays, which was the time when there were more patients with many nursing activities such as examinations and procedures performed. These clinical factors might have influenced the adherence to SPs in this study.

This study showed that the observed adherence was associated with work experience and work department in quantile regression analysis and patient safety climate in correlation analysis. Regarding work experience, the shorter the experience, the lower the adherence to SPs in this study. These findings were consistent with a study by Murray, Sundin, and Cope [36], which reported a theory–practice gap in SPs in newly graduated nurses. This gap has been attributed to difficulties newly graduated nurses experience in managing the pressure of limited time compared to experienced nurses working under the same conditions [36, 37]. When newly graduated nurses must take care of many patients, they cannot adequately comply with SPs. Therefore, to improve adherence to SPs, nurse managers should set workloads considering both each nurse’s competency and working conditions.

The work department was also an influencing factor in the observed adherence to SPs in this study. Nurses in the general ward showed the lowest adherence to SPs. Nurses in integrated care units are in charge of eight patients, whereas nurses in general wards are in charge of more than 15 patients [38]. The adherence of nurses in the integrated unit and that of ICU nurses showed no significant difference. However, the adherence of general ward nurses was significantly lower than that of ICU nurses. Thus, organizational factors such as nurse staffing and work environment influenced the performance of individual nurses. Hence, insufficient resources such as staff and facilities might have contributed to the low adherence in the general ward. This result appears to be consistent with findings of other investigations, showing that patients in understaffed units are more likely to develop HAIs [39, 40]. HAIs are nursing-sensitive outcomes [40]. If structure indicators such as adequate nurse staffing levels are not met, process indicators such as adherence to SPs will inevitably not be met at adequate levels [41]. Consequentially, understaffing conditions and the insufficient provision of nursing services lead to increases of HAIs. Therefore, setting an adequate nurse staffing level should take precedence to ensure adequate adherence to SPs.

In this study, we used quantile regression to investigate factors associated with adherence to SPs. Through quantile regression, we identified influencing factors that differed according to the performance level. In the 25% quantile, work experience was a significant factor. This means that clinical nurses need to be prepared to perform clinical practice. Several studies have also supported the need to enhance work readiness during early stages of a nurse’s career due to increased complexity of care and acutely ill patients [42]. In the 50% quantile, the work department, which also determined how many patients a nurse was in charge of, was a significant factor. Compared to ICU nurses, nurses in the general ward showed statistically low adherence to SPs, while nurses in other departments such as the emergency room and integrated care unit did not show a significant difference in adherence to SPs. Some studies have reported that integrated care units in South Korea have a positive effect on patient-centered outcomes such as falls and pressure ulcers [43, 44]. In the integrated care unit, not only falls and pressure ulcers, but also nurses’ adherence to SPs could be better than those in the general ward. Moreover, as better adherence to SPs would ultimately improve final outcomes, an integrated nursing care system should show positive effects on reducing HAIs. There were no significant factors affecting adherence to SPs in the 75% quartile because we could not include all variables related to SP adherence. In addition, most nurses showed very high adherence to SPs.

We did not include knowledge, awareness, or safe environment in the quantile regression model because these three variables did not show significant correlations with observed adherence in this study. However, among them, awareness and safe environment were significantly correlated with self-reported adherence. Several researchers have insisted that knowledge and awareness of hospital infection control are significantly associated with performance [21, 22] and suggested relevant training for clinical nurses for this reason. Thus, increasing knowledge and awareness is a facilitating factor in improving adherence to SPs. However, to improve adherence to SPs, not only facilitators, but also barriers should be considered. In this study, knowledge was not significantly correlated with adherence to SPs in survey or surveillance scores, which was a different result from other studies [19, 28]. The discrepancy might be attributed to the instrument used in this study. In this study, the average score for knowledge was high and the variance was small, indicating little difference between nurses. Thus, further research is needed to revise the knowledge instrument.

## Limitations

This study has some limitations. First, as we assumed that the observed adherence might be distorted due to external factors, we could not observe each behavior in the developed instrument several times. In addition, one nurse was watched by only one observer. Therefore, the possibility of an error by the observer could not be excluded. The possibility of variance in adherence according to the clinical situation could not be considered either. Despite efforts to avoid observer effects, there might be an external effect compared to video surveillance. Second, as the observational survey was conducted only during the day shift on weekdays, we could not investigate changes in adherence to SPs depending upon the shift or various working environments. In addition, three observers participated in the observational study. To reduce discrepancies in results between observers, education and training were conducted several times before observations. However, there might have been differences between observers because one observer observed one nurse. Lastly, as we conducted this study at only one general hospital, we could not consider various organizational factors. This also limited the generalizability of our study results.

## Conclusions

A great difference in adherence to SPs was found between survey and surveillance methods. The observed adherence was lower than the self-reported adherence. However, the observed adherence was significantly correlated with the self-reported adherence. Factors influencing adherence to SPs were organizational factors such as understaffing and work experience rather than knowledge and awareness in the 25% and 50% quantile groups. Clinical nurses in Korea display competency by showing a high willingness to comply with SPs. Thus, an organizational environment needs to be established so that clinical nurses with high competency can comply with SPs in clinical settings. Setting adequate nurse staffing levels and developing strategies to improve competencies for newly graduated nurses are needed to improve adherence to SPs by clinical nurses.

## Data Availability

The data sets used and analyzed during this study can be obtained from the corresponding author upon reasonable request.

## Abbreviations

SPs: Standard Precautions
HAI: Healthcare-Associated Infection
MERS: Middle East Respiratory Syndrome
WHO: World Health Organization
PPE: Personal Protective Equipment
CDC: Center for Disease Control and Prevention
ICU: Intensive Care Unit
OLS: Ordinal Least Square
QR: Quantile
